# Expression and clinical value of circRNAs in serum extracellular vesicles for gastric cancer

**DOI:** 10.3389/fonc.2022.962831

**Published:** 2022-08-17

**Authors:** Ke Xiao, Shirong Li, Juan Ding, Zhen Wang, Ding Wang, Xiangting Cao, Yi Zhang, Zhaogang Dong

**Affiliations:** ^1^ Department of Clinical Laboratory, Qilu Hospital of Shandong University, Jinan, China; ^2^ Department of Laboratory Medicine, Weifang People’s Hospital, Weifang, China

**Keywords:** gastric cancer, circRNA, extracellular vesicle, diagnosis, biomarker

## Abstract

**Objective:**

At present, there are still no effective diagnosis methods for gastric cancer (GC). Increasing evidences indicate that Extracellular Vesicle circular RNAs (EV circRNAs) play a crucial role in several diseases. However, their correlations with GC are not clarified. This study aims to investigate the expression profile of serum EV circRNAs in GC and evaluate its potential clinical value.

**Methods:**

High-throughput RNA sequencing (RNA-seq) was used to assess circRNA expression profiles between 4 patients with GC and 4 healthy controls. Gene Ontology (GO) and Kyoto Encyclopedia of Genes and Genomes (KEGG) pathway enrichment analyses were employed to determine the biological functions of differentially expressed (DE) circRNAs. A circRNA-miRNA-mRNA network was constructed using bioinformatics tools. Reverse transcription-quantitative polymerase chain reaction (RT-q)PCR was used to validate the dysregulated circRNAs. Receiver operating characteristic (ROC) curves were used to evaluate the diagnostic value of circRNAs for GC.

**Results:**

A total of 4692 circRNAs were detected in the serum EVs of healthy controls and patients with GC, most of which were novel (98%) and intergenic (52%). 7 circRNAs were upregulated and 4 circRNAs were downregulated (|log_2_Fold Change| > 2, *P <* 0.05). GO and KEGG pathway enrichment analyses revealed that DE circRNAs were primarily involved in glutathione metabolism, protein folding, and drug metabolism-cytochrome P450. Of these, 3 circRNAs (Chr10q11, Chr1p11, and Chr7q11) were identified to be significantly overexpressed in patients with GC compared with healthy controls using RT-qPCR. The combination of 3 EV circRNAs and carcinoembryonic antigen (CEA) produced an area under the curve (AUC) of 0.866 (95%CI: 0.803-0.915) with a sensitivity and specificity of 80.4% and 81.8%, respectively. Additionally, the expression levels of 3 EV circRNAs were significantly correlated with tumor size, lymph node metastasis, and TNM stage. The circRNA-miRNA-mRNA network showed that the 3 identified circRNAs were predicted to interact with 13 miRNAs and 91 mRNAs.

**Conclusion:**

Our results illustrate that the panel of EV circRNAs in serum are aberrantly expressed and may act as the suitable biomarkers for gastric cancer.

## Introduction

Gastric cancer (GC) is the fifth most common type of cancer and the third most common cause of cancer-related death globally. Global cancer statistics show more than one million new cases are diagnosed every year (1–3). Due to the lack of effective markers for GC, the 5-year survival rate is more than 90% in the early stages. Whereas it is only 5-20% in patients with advanced stage, and the median overall survival (OS) is 10 months (1, 4, 5). Therefore, early detection of GC is particularly important for improving the survival rates (6, 7). At present, the diagnostic methods of GC include endoscopic ultrasonography, tissue biopsy, imaging examination, and screening of tumor markers and so on (8). But these methods still have defects to some extent. For example, tissue biopsy is invasive, complicated, and expensive. Improper preparation of samples can lead to misdiagnosis or missed diagnosis (9). X-ray and CT scans have difficulty in detecting lesions less than 1cm (10). The traditional tumor markers for GC detection include carcinoembryonic antigen (CEA), carbohydrate antigen 19−9 (CA19−9), and carbohydrate antigen 72−4 (CA72−4), but they are insufficient (11). For example, our previous study has showed that the expression of CA19-9 and CA72-4 do not differ between groups (12). Therefore, identifying novel markers for GC remains a challenge.

Circular RNAs (circRNAs) are a unique class of non-coding RNAs (ncRNAs) that form a covalently closed loop-structure without 5’ and 3’ ends, making it resistant to RNase R digestion (13, 14).. CircRNAs can be divided into exon, intron and exon-intron according to its origin. It is abundant, stable, highly conserved and tissue-specific (15). As circRNAs do not typically encode protein and its function is not clear, they are initially considered to be the by-products of post-transcriptional modification of mRNA (16). Recently, circRNAs can be detected in body fluid, serum, plasma, and tissue samples, and play an important regulatory role in the development of most diseases (17). Therefore, they have the potential to become promising biomarkers. Chi Hin Wong et al. found that circRTN4 was significantly upregulated in primary tumors from pancreatic ductal adenocarcinoma patients (18). The levels of plasma circFARSA in lung cancer patients was higher than that in healthy controls, suggesting that circFARSA may be used as a potential biomarker for non-invasive diagnosis (19). Three circRNAs (hsa_circ_0000370, hsa_circ_0082812 and hsa_circ_0035445) were abnormally expressed in the plasma of colorectal cancer patients, with an area under the curve (AUC) of 0.815, 0.737 and 0.703, respectively (20). These results suggest that circRNAs can be served as a non-invasive biomarker for cancer diagnosis. Zhu Jin et al. found that the absence of circ-BIRC6 abrogated the progression of non-small cell lung cancer by targeting miR-4491 (21). A study from Zong et al. (22) showed that the expression of circRNA_102231 was significantly upregulated in lung adenocarcinoma and was related to lymph node metastasis. Chen et al. (23) found that patients with lower expression levels of circRNA cRAPGEF5 had a poorer prognosis and inhibit the proliferation and migration of renal cell carcinoma through miR-27a-3p/TXNIP pathway. Interesting, circRNAs also play an indispensable role in GC. A study showed that the upregulated expression of circPVT1 in GC tissues competitively bound to miR-125, promoting the proliferation of cancer cells (24). Zang et al. found that the downregulated expression of circEIF4G3 was observed in GC and was associated with poor clinical outcomes (25). Another study showed that circMAN1A2 could regulate MTA2 through sponging miR-1236-3p, promoting the progression of H. pylori-induced GC (26). These above studies give us a hint that the value of circRNAs in GC remains to be further clarified.

Accumulating studies have shown that extracellular vesicles (EVs) are important carriers of circRNAs. The protective effects of EV membranes and the unique structure of circRNAs extend the half-life of circRNAs and increase RNase R resistance compared to linear RNAs (27, 28). A study showed that there were significant differences between hsa-circRNA-0005795 and hsa-circRNA-0088088 in serum exosomes (29). Chen et al. (30) found that serum exocrine circPRMT5 was increased in bladder cancer, which was closely related to stage and prognosis, and a MiR-30c/SNAIL1/E-cadherin pathway was involved in the process of tumor EMT. Some studies have explored its value for biomarkers. For example, Lu et al. (31) observed that the high expression of circ-RanGAP1 was closely related to stage, metastasis, and prognosis. EV circRNAs have provided a novel perspective for GC diagnosis. However, the research on EV circRNAs as tumor marker in GC are considerably limited.

There are difficulties in detecting and identifying novel EVs circRNAs. In the past decade, RNA-seq has become an indispensable method for transcriptome analysis (32, 33). And now it is most commonly method in analyzing differentially expressed (DE) genes. Zhu et al. (34) performed high-throughput RNA-seq and identified 84 circular RNAs, 41 miRNAs and 398 mRNAs in CRC. Long et al. (35) found that the expression of hsa_circ_0007694 in thyroid papillary carcinoma was downregulated through high-throughput sequencing. Further study found that PI3K/AKT/mTOR and Wnt signaling pathway were involved. In fact, RNA-seq technology can be used to identify novel biomarkers for improving tumor diagnosis. However, there are few studies on EV circRNAs in GC.

In the current study, RNA-seq technology was used to determine the expression profiles of circRNAs in serum EV between patients with GC and healthy controls. GO and KEGG pathway analyses indicated that circRNAs may serve as potential therapeutic targets in GC. CeRNA networks showed a potential role of the circRNAs in cancer progression. The expression and diagnostic value of 3 candidate circRNAs were further evaluated. In addition, the association of these 3 circRNAs with clinicopathological variables was assessed.

## Materials and methods

### Patients and control subjects

A total of 260 serum samples were collected from Qilu Hospital of Shandong University from December 2016 to December 2017. Of these, serum samples of 4 patients with GC and 4 healthy controls were used for high-throughput sequencing. Subsequently, the remaining 252 samples were randomly assigned to training set (10 GC and 10 healthy controls) and validation set, including 44 healthy controls (age ranged 29-66years), chronic gastritis (age ranged 32-88 years), typical hyperplasia (age ranged 33-76 years), and GC (age ranged 25-82 years). Participants’ demographics and clinicopathological parameters were also recorded. The inclusion criteria were the same as our previous study (36). This study has been approved by the Medical Ethics Committee of Qilu Hospital of Shandong University (KYLL-2015-097).

### Extraction of extracellular vesicles

The extraction of extracellular vesicles were performed as described in our previous study (12, 36).

### High-throughput sequencing

Total RNAs were extracted from serum EVs, and then Ribo-Zero rRNA Removal (Illumina, USA) was used to deplete ribosomal RNA. RNA libraries were constructed using TruSeq Stranded Total RNA Library Prep Kit (Illumina). BioAnalyzer 2100 system (Agilent Technologies, Santa Clara, CA, USA) was used to quantify and quality-control libraries. Then, 10 pM libraries were denatured to single-stranded DNA molecules, captured on Illumina flow cells, amplified *in situ* as clusters, and sequenced for 150 cycles using an Illumina HiSeq platform.

### CircRNAs sequencing data analysis

Briefly, paired-end reads were harvested from Illumina HiSeq 4000 platform and controlled quality by Q30. After 3’ adaptor-trimming and low-quality read were removed by cutadapt software (v1.9.3), the high-quality reads were aligned to the reference genome/transcriptome with STAR software. CircRNAs were detected and identified with DCC software. EdgeR software was used to normalize the data and perform DE circRNA analysis.

### Gene Ontology and Kyoto Encyclopedia of Genes and Genomes pathway analysis

To predict the function of circRNAs, 20 miRNAs with the strongest binding to DE circRNAs, and its target gene 50 mRNAs were selected for GO and KEGG pathway analysis. P-value< 0.05 was considered as the threshold of significant enrichment. The differential genes were grouped into hierarchical categories of molecular function, biological process, and cellular component based on the Gene Ontology database (http://www.geneontology.org/). Pathway analysis was performed to assess the potential pathways of the differentially expressed genes according to KEGG (http://www.genome.jp/kegg/).

### Prediction of circRNA-miRNA-mRNA interactions

We predicted the miRNA-binding sites of circRNA using two different algorithms: TargetScan and miRANDA based on database from miRBase release 21.0. MiRNA-mRNA regulatory relationships was identified using miRTarBase database. Cytoscape was applied to draw the graph of a circRNA-miRNA-mRNA interaction network.

### RNA extraction and quantitative real-time RT-PCR

Serum EVs RNA was extracted using exoRNeasy Serum/Plasma Midi Kit (Cat:77044, Qiagen, Germany) according to the manufacturer’s instructions. ND-1000 Nanodrop (Thermo Fisher, USA) was applied to test the purity of total RNA. All-in-one first-strand cDNA synthesis kit (Cat: QP006, GeneCopoeia company, Rockville, Maryland) was used to reversely transcribe total RNA to cDNA according to instructions. cDNA was diluted at 1:5. PCR reactions were performed using CFX96 (BIO-RAD, USA) with All-in-one™ qPCR Mix (Cat: QP001, GeneCopoeia company). Amplification cycle conditions were as follows: an initial incubation at 95°C 10min, then 95°C 15s, 62°C 20s, 72°C 10s, and the cycle was 40. The sequences of primers are listed in **Table 1**. Data was normalized using 18S, and relative expression levels were evaluated using the 2^−ΔΔCT^ method.

**Table 1:** Primer sequences of PCR.

CircRNAID	primer type	primer sequence	Amplicon length, bp
XLOC_000992 (chr1:121484069-121485087-)	Forward	CAACTCTGTCAGTTGAATACACAC	463
	Reverse	GTCTGTAAGTGGATATTCTGACATC	
Chr1p11 (chr1:121485002-121485340+)	Forward	TTCAGCCGCTTTGAGGTC	196
	Reverse	CCAACGAAGGCCACAAGA	
Chr10q11 (chr10:42385521-42396165-)	Forward	TCGGTTCCATTTGATGATGA	214
	Reverse	CGAATGGAATGGAATGGAA	
chr4:49282921-49307540+	Forward	GAATCTCCAGAGGAGGACACG	231
	Reverse	CTCAAGATTCATGCCTGCCTCAC	
chr6:58778997-58779502+	Forward	GCAATGGGATATTTGGACTTCTTTG	253
	Reverse	GTTTCTGAGATTGCTTCCGTCTAG	
GSTA1 (chr6:52650927-52691423+)	Forward	AAAGCCAATGGCATCAACA	169
	Reverse	TCCATGTCTGCAGTTGCTTC	
Chr7q11 (chr7:61967596-61970486+)	Forward	TTTGAGGCCTTCGTTGGA	192
	Reverse	CCCTTTCCACTGTTGGCA	
RP11-351A11.1 (chr6:119261993-119262320+)	Forward	CTTCTCCAGCTCCAAGCAGC	163
	Reverse	CAGCCAGGCTTGCATCCTTC	
XLOC_13994 (chr21:9827153-9827349+)	Forward	CCCGTTTCCAACGAAGG	164
	Reverse	GGCTGCATTCCACACACA	
UNC45B1 (chr17:33478113-33478328-)	Forward	ATGGCGAGTGAACAGGGA	156
	Reverse	GGTCGCCACGTCTGATCT	
UNC45B2 (chr17:33478158-33478373-)	Forward	TTTAAGCATATTTGTCAGCGGAG	132
	Reverse	GTCTTCCGTACGCCACATTTC	
18S	Forward	AGGGACAAGTGGCGTTCAGC	67
	Reverse	CGGACATCTAAGGGCATCAC	
GAPDH	Forward	TGCACCACCAACTGCTTAGC	87
	Reverse	GGCATGGACTGTGGTCATGAG	

### Fecal occult blood tests

Bleeding of digestive tracts was measured by the Colloidal gold-based fecal occult blood diagnostic Kit (Chemtron Biotech Co. Shanghai, China).

### Statistical analysis

Kolmogorov Smirnov test was used to determine the distribution of data in each group. Data was presented as median and interquartile ranges. Serum EV circRNAs levels were evaluated using Mann Whitney U or Kruskal Wallis test in different groups, including clinicopathological variables. ROC curves were used to evaluate the diagnostic value of the potential biomarkers in GC. The cut-off value was calculated according to Youden index (sensitivity + specificity-1). *P*<0.05 was considered statistically significant. SPSS 25.0 (Chicago, Illinois, USA) and Medcalc 8.0 (Korea) were used in this study.

## Results

### Characteristics of the total circRNAs expression

**Figure 1:**
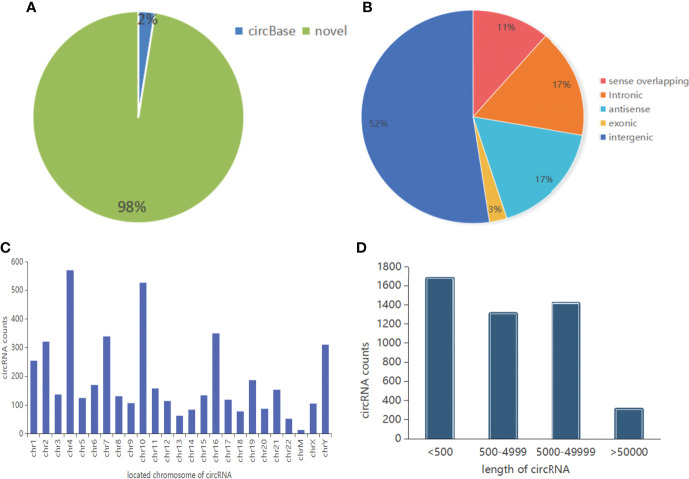
The characteristics of circRNAs. **(A)** The classification of circRNAs identified by sequencing. **(B)** The categories of circRNAs. **(C)** The location distribution of circRNAs on chromosomes. **(D)** The length distribution of circRNAs.

The A260/280 ratio range of EVs RNA of 4 healthy controls and 4 GC were 1.8-2.1. This indicated that the purity of the extracted RNA was high and could be used for high-throughput sequencing (**Table 2**). Results showed that a total of 4692 circRNAs (reads ≥ 2 in a single sample) were identified, of which 4575 (98%) were novel, and 117 (2%) have been reported in circBase database (**Figure 1A**). According to the location of their parent genes, circRNAs were divided into five categories. Intergenic, intronic, antisense, sense overlapping, and exonic accounted for 52%, 17%, 17%, 11%, 3%, respectively (**Figure 1B**). In addition, circRNAs were observed to be widely distributed to all chromosomes according to the location of its transcriptional gene (**Figure 1C**). Further analysis demonstrated that the length of circRNAs was predominantly < 5,000 bp (**Figure 1D**).

**Table 2:** RNA quality inspection of serum extracellular vesicles.

Sample ID	Sample Name	Conc. (ng/µl)	Volume (µl)	Quantity (ng)	QC Results
1	HC1	2.070	10	20.700	Pass
2	HC2	4.200	10	42.000	Pass
3	HC3	2.200	10	22.000	Pass
4	HC4	2.270	10	22.700	Pass
5	GC1	9.020	10	90.200	Pass
6	GC2	2.200	10	22.000	Pass
7	GC3	4.620	10	46.200	Pass
8	GC4	2.870	10	28.700	Pass

For spectrophotometer, the O.D. A260/A280 ratio should be close to 2.0 for pure RNA (ratios between 1.8 and 2.1 are acceptable).

HC, healthy control; GC, gastric cancer.

### Differential circRNA profiles in serum extracellular vesicles in patients with GC and healthy controls

**Figure 2:**
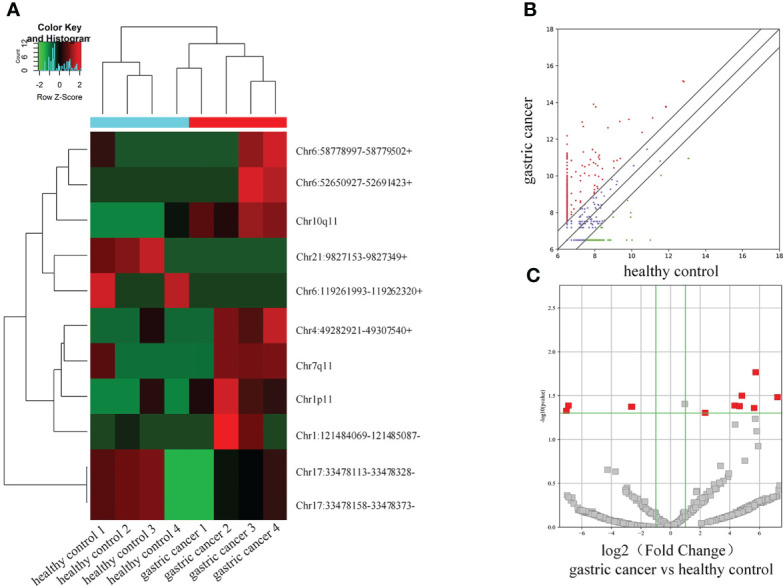
Differential circRNA expression profiles in serum extracellular vesicles. **(A)** Cluster heat map. **(B)** Scatter map. **(C)** Volcano map.

11 differential circRNAs were initially selected according to the criteria |log_2_Fold Change| > 2, *P <* 0.05). 7 were significantly upregulated and 4 were downregulated (**Table 3**). A cluster heat map (**Figure 2A**), scatter map (**Figure 2B**), and volcano map (**Figure 2C**) were used to visualize the differences of circRNAs in GC and healthy controls.

**Table 3:** Differentially expressed circRNAs in gastric cancer and healty control.

GENE ID	CircRNAID	logFC	PValue	FDR	regulation	chrom	txStart	txEnd	strand	source	best_transcript	GeneName	Catalog	predicted_sequence_length
XLOC_000992	chr1:121484069-121485087-	7.2302627	0.032888	1	up	chr1	1.2E+08	1.2E+08	–	novel	TCONS_00001651	XLOC_000992	sense overlapping	1019
**Chr1p11**	chr1:121485002-121485340+	5.748942	0.0171205	1	up	chr1	1.2E+08	1.2E+08	+	novel	TCONS_00001651	XLOC_000992	antisense	339
**Chr10q11**	chr10:42385521-42396165-	5.6448719	0.0437104	1	up	chr10	4.2E+07	4.2E+07	–	novel	TCONS_00017955	XLOC_008785	sense overlapping	10645
	chr4:49282921-49307540+	4.8211342	0.0316848	1	up	chr4	4.9E+07	4.9E+07	+	novel			intergenic	24620
	chr6:58778997-58779502+	4.6582758	0.0416758	1	up	chr6	5.9E+07	5.9E+07	+	novel			intergenic	506
GSTA1	chr6:52650927-52691423+	4.3320003	0.040987	1	up	chr6	5.3E+07	5.3E+07	+	novel	NM_145740	GSTA1	antisense	40497
**Chr7q11**	chr7:61967596-61970486+	2.3361354	0.0495793	1	up	chr7	6.2E+07	6.2E+07	+	novel			intergenic	2891
RP11-351A11.1	chr6:119261993-119262320+	-7.0399857	0.0470991	1	down	chr6	1.2E+08	1.2E+08	+	novel	ENST00000518570	RP11-351A11.1	intronic	328
XLOC_13994	chr21:9827153-9827349+	-6.8988159	0.0409665	1	down	chr21	9827152	9827349	+	novel	TCONS_00029068	XLOC_013994	antisense	197
UNC45B1	chr17:33478113-33478328-	-2.646774	0.0421989	1	down	chr17	3.3E+07	3.3E+07	–	novel	NM_173167	UNC45B	antisense	216
UNC45B2	chr17:33478158-33478373-	-2.6093865	0.0422676	1	down	chr17	3.3E+07	3.3E+07	–	novel	NM_173167	UNC45B	antisense	216

### GO enrichment analysis of DE circular RNA

**Figure 3:**
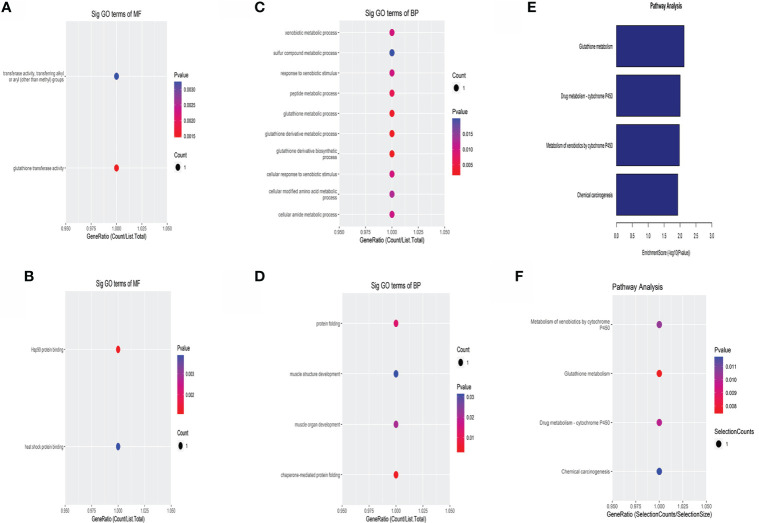
GO and KEGG analysis of the differentially expressed circRNAs. GO analysis of the upregulated **(A)** and the downregulated **(B)** DE circRNAs in terms of molecular function. GO analysis of the upregulated **(C)** and the downregulated **(D)** DE circRNAs in biological processes. KEGG enrichment analysis of the differentially expressed circRNAs **(E, F)**. X-axis represented GeneRatio, and it equals the ratio of the number of DE genes associated with the gene ontology term to the total number of DE genes.

The enrichment results of molecular function (MF) showed that the upregulated circRNAs were mainly related to glutathione transferase activity and transferase activity (**Figure 3A**). The major downregulated circRNAs were associated with heat shock protein (HSP) binding and Hsp90 protein binding (**Figure 3B**). In the biological process (BP), the upregulated circRNAs were involved in glutathione derivative metabolic process and glutathione derivative biosynthetic process (**Figure 3C**). The downregulated circRNAs were primarily enriched in chaperone-mediated protein folding and protein folding (**Figure 3D**).

### KEGG analysis of DE circRNAs

The results of KEGG were shown in a column chart (**Figure 3E**) and bubble chart (**Figure 3F**). DE circRNAs were enriched in glutathione metabolism, drug metabolism-cytochrome P450, metabolism of xenobiotics by cytochrome P450, and chemical carcinogenesis.

### Preliminary screening of DE circRNAs in training set

**Figure 4:**
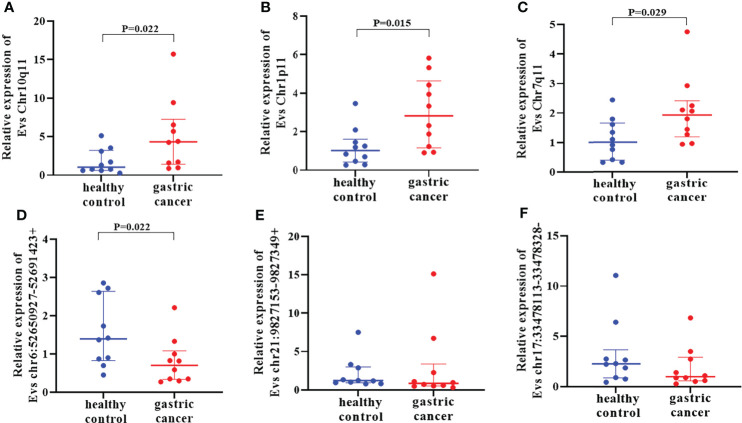
Verification of differentially expressed circRNAs in training set. The expression of 6 EVs circRNAs Chr10q11 **(A)**, Chr1p11 **(B)**, Chr7q11 **(C)** chr6:52650927-52691423+ **(D)**, chr21:9827153-9827349+ **(E)** and chr17:33478113-33478328- **(F)** between healthy controls and gastric cancer.

First of all, 11 DE circRNAs were verified in training set including 10 healthy controls and 10 GC using RT-qPCR. Results showed that the expression levels of circRNA Chr10q11, Chr1p11, Chr7q11 in GC were significantly higher than those in healthy controls (**Figures 4A–C**) (all *P* < 0.05). Whereas circRNA chr6:52650927-52691423+ was lower in GC (*P* = 0.022) (**Figure 4D**). Interesting, circRNA chr21:9827153-9827349+ and chr17:33478113-33478328- were not significantly different between two groups (**Figures 4E, F**). The remaining 5 circRNAs were not amplified or nonspecific amplification. Remarkably, the expression of circRNA chr6:52650927-52691423+ in training set was inconsistent with the results of RNA-seq, and it was not considered in the following experiments.

### CircRNAs feature verification

The 3 DE circRNAs, Chr10q11, Chr1p11, and Chr7q11, were amplified by RT-qPCR, and PCR products were identified using Sanger sequencing. Results showed that 3 circRNA products had specific sequences of cyclization sites, and the bases were TT, AC, and TG, respectively (**Supplementary Figures 1A–C**). To further observe the stability of 3 circRNAs, RT-qPCR was used to detect total RNA samples treated with or without RNase R, respectively. Results showed that after RNase R digestion, the expression levels of circRNAs did not differ significantly, but linear RNA GAPDH were significantly downregulated (**Supplementary Figures 2**, P=0.01).

### DE circRNAs in validation set

**Figure 5:**
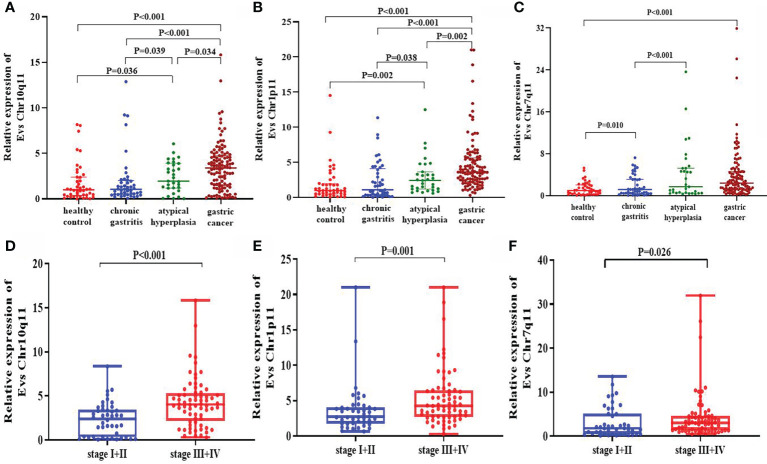
Expression of differentially expressed circRNAs in validation set. The expressions of circRNA Chr10q11 **(A)**, Chr1p11 **(B)**, and Chr7q11 **(C)** in different groups including healthy control, chronic gastritis, atypical hyperplasia and gastric cancer. Meanwhile, the expression of circRNA Chr10q11 **(D)**, Chr1p11 **(E)**, Chr7q11 **(F)** in stage I + II and stage III + IV were also compared.

In validation set, the expression of circRNA Chr10q11 in GC [3.395 (1.455-4.743)] was higher than that in healthy controls [1.000 (0.378-2.395)], chronic gastritis [1.060 (0.495-2.025)] and atypical hyperplasia [1.950 (1.180-3.900)], respectively. Atypical hyperplasia was higher than that of healthy controls and chronic gastritis (**Figure 5A**). Similarly, the expression of circRNA Chr1p11 in GC [3.600 (2.325-5.625)] was higher than that in healthy controls [0.995 (0.495-1.965)], chronic gastritis [1.100 (0.325-4.105)] and atypical hyperplasia [2.430 (1.200-3.660)], respectively. Chr1p11 was higher in atypical hyperplasia than in healthy controls and chronic gastritis (**Figure 5B**). **Figure 5C** showed that circRNA Chr7q11 in GC [2.403 (1.225-4.639)] was higher than that in healthy control [1.001 (0.279-2.061)], chronic gastritis [1.183 (0.419-3.130)] and atypical hyperplasia [1.721 (0.520-5.247)]. Additionally, we investigated the expression of circRNAs in the different TNM stages. The levels of serum EVs circRNA Chr10q11 (**Figure 5D**), Chr1p11 (**Figure 5E**) and Chr7q11 (**Figure 5F**) in stage III + IV were all significantly higher than that in stage I + II (all *P* < 0.05).

### Relationship between DE circRNAs and clinicopathological parameters

Results from **Table 4** revealed that tumor size, lymph node metastasis, distant metastasis, and TNM stage were all correlated with 3 circRNAs including Chr10q11, Chr1p11, Chr7q11 (all *P* < 0.05). Sex, age, blood pressure, location, or fecal occult blood were all not related to the above 3 circRANs (all *P* > 0.05). In addition, circRNA Chr10q11 and Chr1p11 were both associated with invasion depth (all *P* < 0.05). But circRNA Chr10q11 was observed to be not significantly correlated with Bormann classification (*P* = 0.035).

**Table 4:** Correlations between 3 DE circRNAs and clinicopathological variables.

Variables	N	Chr10q11		Chr1p11		Chr7q11	
		median (interquartile range)	P value	median (interquartile range)	P value	median (interquartile range)	P value
Gender							
Male	82	3.095 (1.29,4.57)	0.220	3.485 (2.3,5.86)	0.921	2.328 (1.224,4.563)	0.454
Female	30	3.685 (2.77,5.07)		3.64 (2.59,4.4)		3.06 (1.228,5.44)	
Age							
≤61	56	3.5 (1.335,4.56)	0.714	3.55 (2.265,5.01)	0.425	2.177 (1.028,4.278)	0.161
>61	56	3.325 (1.61,5.155)		3.93 (2.415,6.16)		2.747 (1.379,5.357)	
Hypertension							
No	87	3.57 (1.47,4.92)	0.693	3.6 (2.23,5.68)	0.579	2.676 (1.256,5.098)	0.153
Yes	25	3.22 (1.45,4.08)		4.31 (2.51,5.41)		1.787 (0.666,3.681)	
Cancer location							
Antrum	44	3.9 (2.105,4.935)	0.177	3.72 (2.62,4.675)	0.695	2.837 (1.28,5.357)	0.220
Angulus	19	3.36 (0.39,4.08)		3.5 (2.02,4.48)		1.51 (0.901,4.351)	
Cardia	25	2.83 (1.05,4.28)		3 (2.4,6.28)		2.676 (1.146,4.185)	
Body	24	3.48 (1.885,5.13)		4.04 (1.99,6.055)		2.502 (1.829,6.021)	
Tumor diameter							
≤3cm	49	1.61 (0.39,3.36)	<0.001	3.09 (2.01,4.4)	0.005	1.51 (0.614,4.57)	0.005
>3cm	63	4.16 (2.83,5.71)		4.18 (2.71,6.46)		2.989 (1.546,4.64)	
Differentiation							
Well	11	2.77 (0.27,3.77)	0.086	2.65 (2.02,3.84)	0.256	1.535 (0.495,5.098)	0.409
Moderately	23	2.83 (0.49,4.55)		3.39 (1.75,5.68)		2.077 (1.224,9.064)	
Poorly	78	3.685 (1.57,5.11)		3.64 (2.65,5.8)		2.65 (1.256,4.557)	
Bormann type							
I	19	2.77 (0.27,4.29)	0.035	2.91 (1.83,4.48)	0.170	2.077 (0.989,6.279)	0.381
II	46	2.88 (1.16,4.28)		3.55 (2.3,5.37)		1.876 (1.146,4.185)	
III	25	3.71 (2.83,4.75)		5.02 (2.66,6.82)		3.578 (1.224,5.898)	
IV	22	4.4 (2.56,5.12)		4.01 (2.66,5.22)		3.033 (1.382,4.02)	
Invasion depth							
T1	28	2.75 (0.37,3.595)	<0.001	3.075 (1.925,4.04)	0.012	1.921 (0.616,5.71)	0.149
T2	16	1.645 (0.32,3.325)		2.565 (1.435,3.94)		1.656 (0.428,3.696)	
T3	18	4.08 (1.66,4.95)		3.97 (2.77,9)		2.615 (1.36,3.78)	
T4	50	3.95 (2.43,5.77)		4.29 (2.71,5.86)		3.158 (1.451,4.638)	
Lymphatic metastasis							
No	40	2.105 (0.37,3.42)	<0.001	3.375 (1.925,4.355)	0.034	1.54 (0.536,4.834)	0.028
Yes	72	4.045 (2.26,5.385)		3.965 (2.655,6.1)		2.902 (1.416,4.639)	
Distal metastasis							
No	88	2.83 (1.18,4.22)	<0.001	3.485 (2.215,5.195)	0.004	2.176 (1.001,4.604)	0.042
Yes	24	5.125 (3.845,7.245)		4.885 (3.215,10.21)		3.63 (1.948,4.839)	
TNM Stage							
I	32	2.105 (0.26,3.265)	<0.001	2.705 (1.79,3.965)	0.011	1.487 (0.413,4.834)	0.042
II	37	3.58 (1.61,4.72)		3.61 (2.07,5.42)		2.352 (1.256,6.943)	
III	29	3.43 (1.29,4.75)		3.6 (2.66,5.43)		2.869 (1.451,4.351)	
IV	24	5.125 (3.845,7.245)		4.885 (3.215,10.21)		3.63 (1.948,4.839)	
Fecal Occult Blood							
No	81	3.58 (1.68,4.95)	0.108	3.67 (2.43,5.8)	0.554	2.638 (1.228,5.124)	0.27
Yes	31	2.96 (0.87,4.55)		3.47 (2.2,5.22)		2.352 (0.989,4.02)	

### Diagnostic value of DE circRNAs

**Figure 6:**
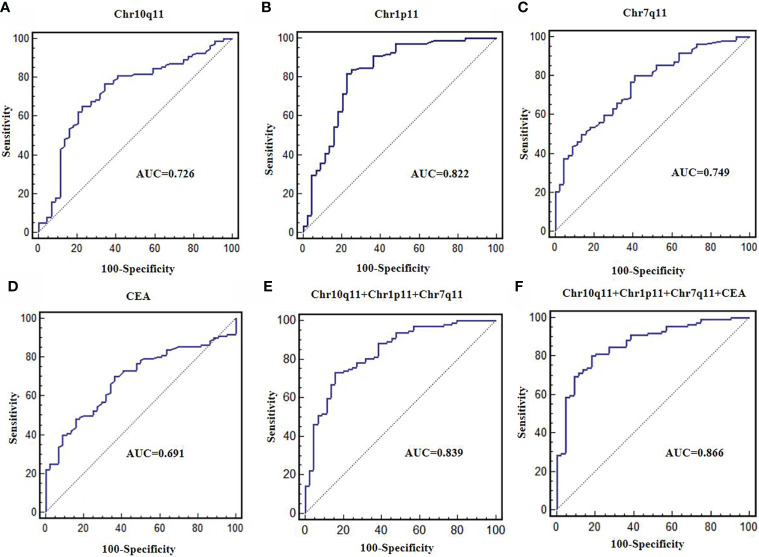
Diagnostic value of differentially expressed circRNAs. ROC curves were used to evaluate the diagnostic value of circRNA Chr10q11 **(A)**, Chr1p11 **(B)**, Chr7q11 **(C)**, CEA **(D)**, 3 circRNAs combination **(E)**, 3 circRNAs + CEA **(F)**.

The AUC of circRNA Chr10q11, Chr1p11, Chr7q11 and CEA were 0.726, 0.822, 0.749 and 0.691, respectively. The sensitivity and specificity of these above molecular were 76.8% and 65.9%, 82.1% and 77.3%, 79.5% and 59.1%, 70.5% and 63.6%, respectively (**Figures 6A–D**, **Table 5**). The AUC of the combined 3 circRNAs was 0.839 (95%CI: 0.772-0.893) with sensitivity 73.2% and specificity 84.1% (**Figure 6E**). When combining 3 circRANs and CEA, the AUC was 0.866 (95%CI: 0.803-0.915). The sensitivity and specificity were 80.4% and 81.8% (**Figure 6F**; **Table 5**), achieving a high classification power for GC and healthy controls.

**Table 5:** The diagnostic value of Chr10q11, Chr1p11, Chr7q11, and CEA in gastric cancer.

	cutoff	sensitivity (95%CI)	specificity (95%CI)	AUC(95%CI)
Chr10q11	1.330	76.8 ( 67.9- 84.2)	65.9 ( 50.1- 79.5)	0.726 (0.649-0.795)
Chr1p11	2.000	82.1 ( 73.8- 88.7)	77.3 ( 62.2- 88.5)	0.822 (0.753-0.879)
Chr7q11	1.070	79.5 ( 70.8- 86.5)	59.1 ( 43.3- 73.7)	0.749 (0.673-0.815)
CEA	2.100	70.5 ( 61.2- 78.8)	63.6 ( 47.8- 77.6)	0.691 (0.612-0.762)
Chr10q11+Chr1p11+Chr7q11		73.2 ( 64.0- 81.1)	84.1 ( 69.9- 93.3)	0.839 (0.772-0.893)
Chr10q11+Chr1p11+Chr7q11+CEA		80.4 ( 71.8- 87.3)	81.8 ( 67.3- 91.8)	0.866 (0.803-0.915)

### Predicted circRNA-miRNA-mRNA network based on the DE circRNAs

**Figure 7:**
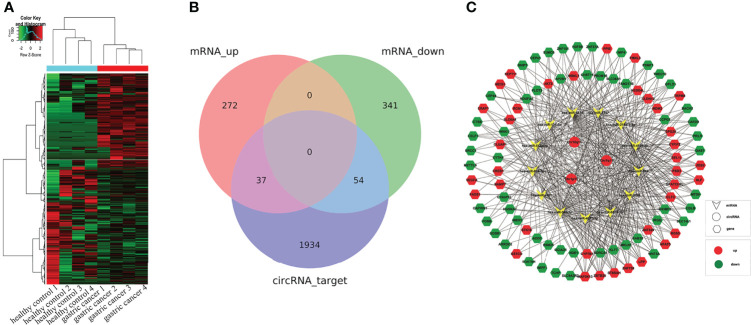
Construction of circRNA-miRNA-mRNA network. **(A)** showed the heatmap of EV mRNAs in sequencing results. **(B)** The intersection of mRNAs from sequencing results and mRNAs from prediction by DE circRNAs. **(C)** Construction of circRNA-miRNA-mRNA network. Circles represent circRNAs, arrows represent miRNAs, and hexagons represent mRNAs.

The 3 selected DE circRNAs, Chr10q11, Chr1p11, and Chr7q11, were used to predict the ceRNA network of circRNA-miRNA-mRNA. First, we analyzed mRNA expression levels in sequencing data (**Figure 7A**). A total of 20,308 mRNAs (13,991 upregulated mRNAs and 6,317 downregulated mRNAs) were obtained. 704 DE mRNAs were screened according to FC > 1.5, *P* < 0.05. Next, TargetScan and miRANDA were used to predict the binding miRNAs, and the top five miRNAs with the strongest binding to each circRNA were selected. Based on this, the top 200 mRNAs with the strongest binding to each miRNA were also predicted. Subsequently, these target genes were intersected with the DE mRNAs in sequencing data. Finally, 37 upregulated mRNAs and 54 downregulated mRNAs were obtained (**Figure 7B**), and then the circRNA-miRNA-mRNA network was drawn. **Figure 7C** showed that DE circRNAs were interacted with 13 miRNAs (hsa-miR-3198, hsa-miR-4677-3p, hsa-miR-450b-3p, hsa-miR-944, hsa-miR-513b-5p, hsa-miR-6888-3p, hsa-miR-580-3p, hsa-miR-219a-1-3p, hsa-miR-4251, hsa-miR-4457, hsa-miR-136-3p, hsa-miR-3174, and hsa-miR-616-3p) and 91 mRNAs. By visualizing the ceRNA network, we can directly observe the center of ceRNA network and the relationship between these components. This provides a reference for future research on the identified circRNAs. In addition, the function of mRNAs were analyzed by GO and KEGG. Results showed that GO (**Supplementary Figures 3A–F**) and KEGG (**Supplementary Figures 4A–D**) were mainly enriched in protein binding, biological regulation, endocytosis, and PI3K-Akt signaling pathway.

## Discussion

With the improvement of living standards and irregular diets, patients with GC is increasing annually. GC has become one of the most common high-incidence malignancies, and over 40% of new cases occur in China (37, 38). Recently, EVs have attracted increasing attention as a novel pathway of intracellular communication. Tumor-derived EVs regulate gastric carcinogenesis and drug resistance by delivering some molecules such as lncRNAs, miRNAs, circRNAs to recipient cells (39). CircRNAs has been proved to be able to participate in the occurrence and development of cancer through competing for miRNAs or *via* other mechanisms (40). Most patients with GC are diagnosed in the first instance with advanced stage, thus the survival rate is low. In this study, 4692 circRNAs were identified, of which 11 were differentially expressed. GO and KEGG analyses of DE circRNAs showed that they were involved in several molecular biological pathways such as glutathione metabolism. Of these, the expression of 3 GC-associated DE circRNA, Chr10q11, Chr1p11, and Chr7q11 were upregulated in GC, and has a good diagnostic value. The construction of a circRNA-miRNA-mRNA network showed 13 miRNA and 91 mRNA interactions. Taken together, our findings provide a new direction for improving the early diagnosis of GC.

EVs are a signaling vector for the exchange of ‘information’ from one cell to another. The cell-derived EVs release into circulation and play a complex role in tumor development, including angiogenesis and inflammation, immunosuppression, and so on (41, 42). Zhang et al. (43) demonstrated that EVs from GC cells induced neutrophil polarization to N2, promoting GC cell migration. EVs are able to function as an intercellular communication medium by various contents (proteins, lipids, DNAs, mRNAs, ncRNAs, etc.). Ye et al. found that hsa_circ_0000069 was significantly upregulated and inhibited the proliferation and migration of human pancreatic ductal epithelial cells by inhibiting STIL expression (44). Studies have shown that EVs are the important carriers of circRNAs. CircRNAs are more stable in serum due to the protection provided by EVs (45). Our results showed that circRNAs were more stable in serum. This provides a basis for EVs circRNAs to be used as biomarkers of GC.

CircRNAs have been found to play roles in several biological processes such as miRNA binding, protein binding, and transcriptional and post-transcriptional regulation. To explore the potential function of circRNAs in this study, we performed GO and KEGG pathway analysis. Results of GO analysis indicated that the most significantly enriched terms were heat shock protein binding and glutathione derivative metabolic process, indicating an association with the basic pathophysiological processes of cancer. It has been shown that the high expression of HSPs is significantly associated with the reduced OS in cancer patients (46). KEGG pathway analysis also revealed that the DE circRNAs were mainly involved in pathways such as glutathione metabolism, drug metabolism-cytochrome P450, and chemical carcinogenesis. The cytochrome P450, a monooxygenase, is the oxidative metabolism of exogenous substances. It has also been found that cytochrome P450 is overexpressed in cancer tissues and can predict the response to chemotherapy (47). This was also consistent with the findings of Luo et al. (48) that higher cytochrome P450 2U1 (CYP2U1) levels were predictive of a poor 5-year OS and were associated with the histopathological grade of BCa. These results suggest that dysregulation of circRNAs in GC may be involved in tumorigenesis.

In clinical practice, the diagnosis of certain tumor-related diseases often requires histopathological confirmation, leading to unnecessary injury. There is an urgent need for simpler and noninvasive diagnostic methods for GC. With the development of liquid biopsy technology, EV circRNAs have become a research hotspot in the field of tumor markers, and has a potential in the diagnosis of various diseases (49, 50). In the current study, serum EV circRNA Chr10q11, Chr1p11, and Chr7q11 expression levels were significantly higher in GC, and related to tumor size, lymph node metastasis, distant metastasis, and TNM stage. Several studies also suggest that EV circRNAs in fluids is associated with pathological features of tumor vascular invasion and TNM stage. Wang et al. found that circ-ITCH was downregulated in serum exosomes, and acted as a sponge of miR-199a-5p, inhibiting proliferation and epithelial-mesenchymal transformation of GC (51). The development of GC is a complex process. The roles of EVs circRNAs in GC need to be further explored.

Patients with GC lack typical symptoms in the early stages, and the sensitivity and specificity of the traditional biomarkers is insufficient. Serum EV circRNAs are easy to be obtained and are widely present in various body fluids, providing a novel approaches for GC diagnosis. Our results showed the AUC of 3 DE circRNAs combined with CEA was 0.866. The sensitivity and specificity were 80.4%, and 81.8%, indicating a better value for GC diagnosis. Previous studies have reported that hsa_circ_0065149, circ-KIAA1244, and hsa_circ_0000419 can be used as tumor markers for GC screening. The sensitivity and specificity are 74.8% and 84% (52–54). These results show that EV circRNAs may help to distinguish patients with GC from healthy controls.

Notably, circRNAs contain miRNA binding sites. It can serve as miRNA sponges to alleviate the inhibitory effects. However, the underlying mechanisms in GC remains unclear. To further explore the potential role of circRNAs in GC, we constructed circRNA-miRNA-mRNA network. Results revealed that the DE circRNAs interacted with 13 miRNAs and 91 mRNAs, which were ultimately involved in the regulation of gene expression. MiRNAs have been confirmed to function by binding to mRNAs. For example, in colon cancer, miR-7 binding to the 3 ′ UTR of TFF3 *via* the PI3K-AKT signaling pathway to inhibit proliferation and migration (55). The abnormally expressed circRNAs may participate in the pathogenesis of GC by regulating miRNAs and their target mRNAs. Hu et al. found that CircPIP5K1A activates KRT80 and PI3K/AKT pathway to promote GC development through sponging miR-671-5p (56). In addition, a study showed that circRAB31, a sponge of mir-885-5p, inhibits GC procession *via* PTEN/PI3K/AKT pathway (57). These results suggest that circRNAs can play a significant role in the occurrence and development of GC through circRNA-miRNA-mRNA networks. However, the present study has still some limitations, including the relatively small sample size and lacking of mechanism research. Further study is guaranteed to determine its clinical predictive value and to elucidate the underlying mechanism in gastric cancer.

## Conclusions

Our findings showed that EVs circRNAs in serum were significantly upregulated in gastric cancer, and related to TNM stage and lymphatic metastasis. It has a good diagnostic value for gastric cancer, which will provide novel insights into early diagnosis for gastric cancer.

## Data availability statement

The datasets presented in this study can be found in online repositories. The names of the repository/repositories and accession number(s) can be found below: https://www.ncbi.nlm.nih.gov/geo/, GSE165394.

## Ethics statement

The studies involving human participants were reviewed and approved by Medical Ethics Committee of Qilu Hospital of Shandong University (KYLL-2015-097). The patients/participants provided their written informed consent to participate in this study.

## Author contributions

KX, ZD and YZ designed the experiments; KX and JD performed the experiments; DW collected the samples; ZW and XC analyzed the data; KX and ZD created the figure and wrote the manuscript; SL and YZ provided comments and suggestions. All the authors reviewed and approved the final version of the manuscript.

## Funding

This research was funded by the National Natural Science Foundation of China (82072358, 81972005, 81572070). Latitudinal research project of Shandong University (6010119015). The Major Scientific and Technological Innovation Project of Shandong Province (2021CXGC010603).

## Conflict of interest

The authors declare that the research was conducted in the absence of any commercial or financial relationships that could be construed as a potential conflict of interest.

## Publisher’s note

All claims expressed in this article are solely those of the authors and do not necessarily represent those of their affiliated organizations, or those of the publisher, the editors and the reviewers. Any product that may be evaluated in this article, or claim that may be made by its manufacturer, is not guaranteed or endorsed by the publisher.

## Supplementary material

Supplementary Figure 1 Sanger sequencing of circRNAs. Sanger sequencing of CircRNAs Chr10q11 **(A)**, Chr1p11 **(B)**, and Chr7q11 **(C)**. The direction of arrows represented splice junction of circRNAs.

Supplementary Figure 2 The effect of RNase R on EV circRNAs. The expression of circRNAs Chr10q11, Chr1p11, Chr7q11 and GAPDH with or without RNase R treatment.

Supplementary Figure 3 GO analysis for mRNA from intersection. The CC, MF, and BP of the upregulated mRNAs **(A–C)** and the downregulated mRNAs **(D–F)**.

Supplementary Figure 4 KEGG analysis for mRNA from intersection. KEGG enrichment analysis of the upregulated mRNAs **(A, B)** and the downregulated mRNAs **(C, D)**.
