# Supplementary figures and images for: Expression and clinical value of circRNAs in serum extracellular vesicles for gastric cancer
Source: Front Oncol. 2022 Aug 17;12:962831. doi: 10.3389/fonc.2022.962831 (PMC9428625; doi:10.3389/fonc.2022.962831)

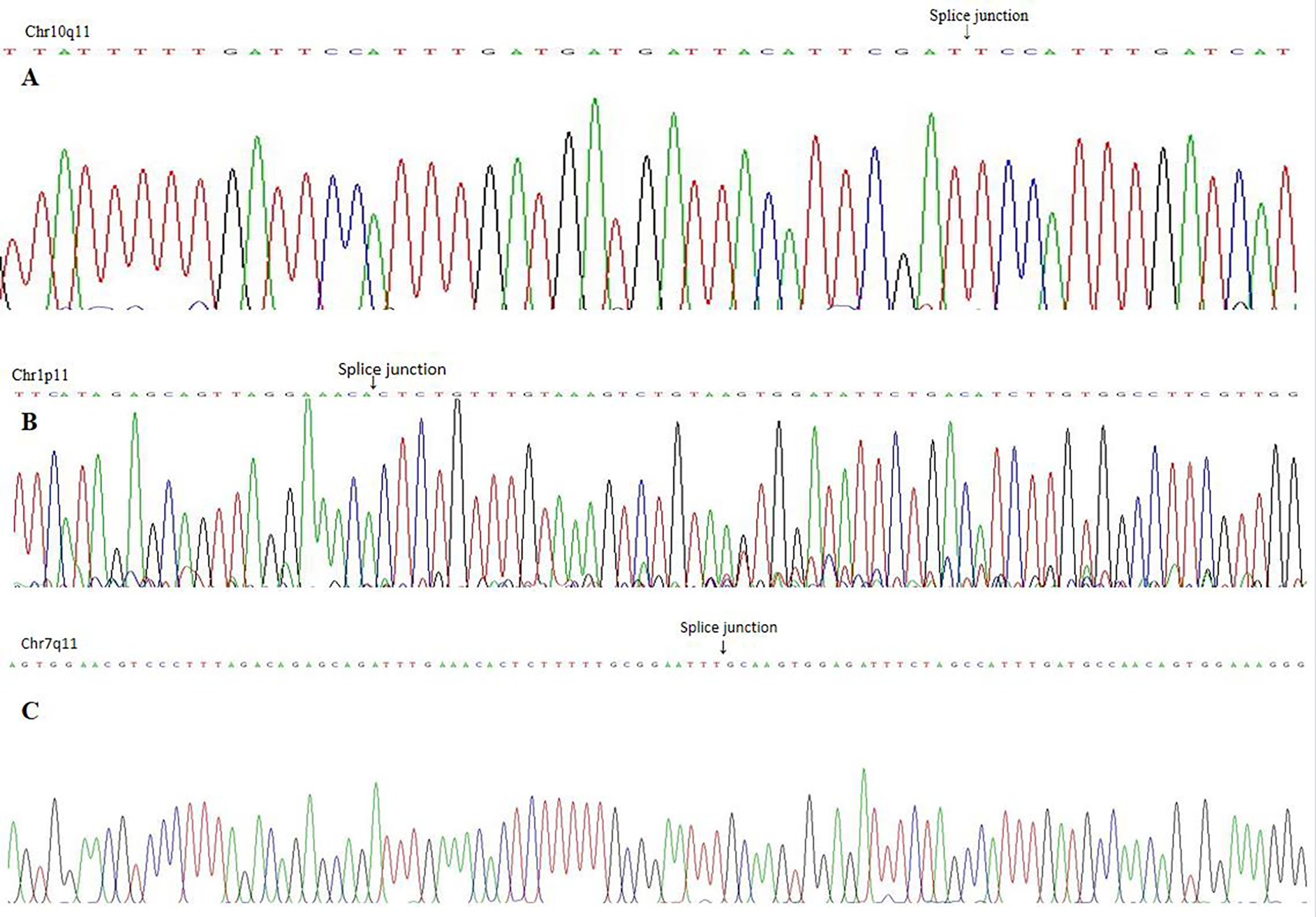

Supplement: Supplementary Figure 1 — Sanger sequencing of circRNAs. Sanger sequencing of CircRNAs Chr10q11 (A), Chr1p11 (B), and Chr7q11 (C). The direction of arrows represented splice junction of circRNAs. [file Image_1.tif]

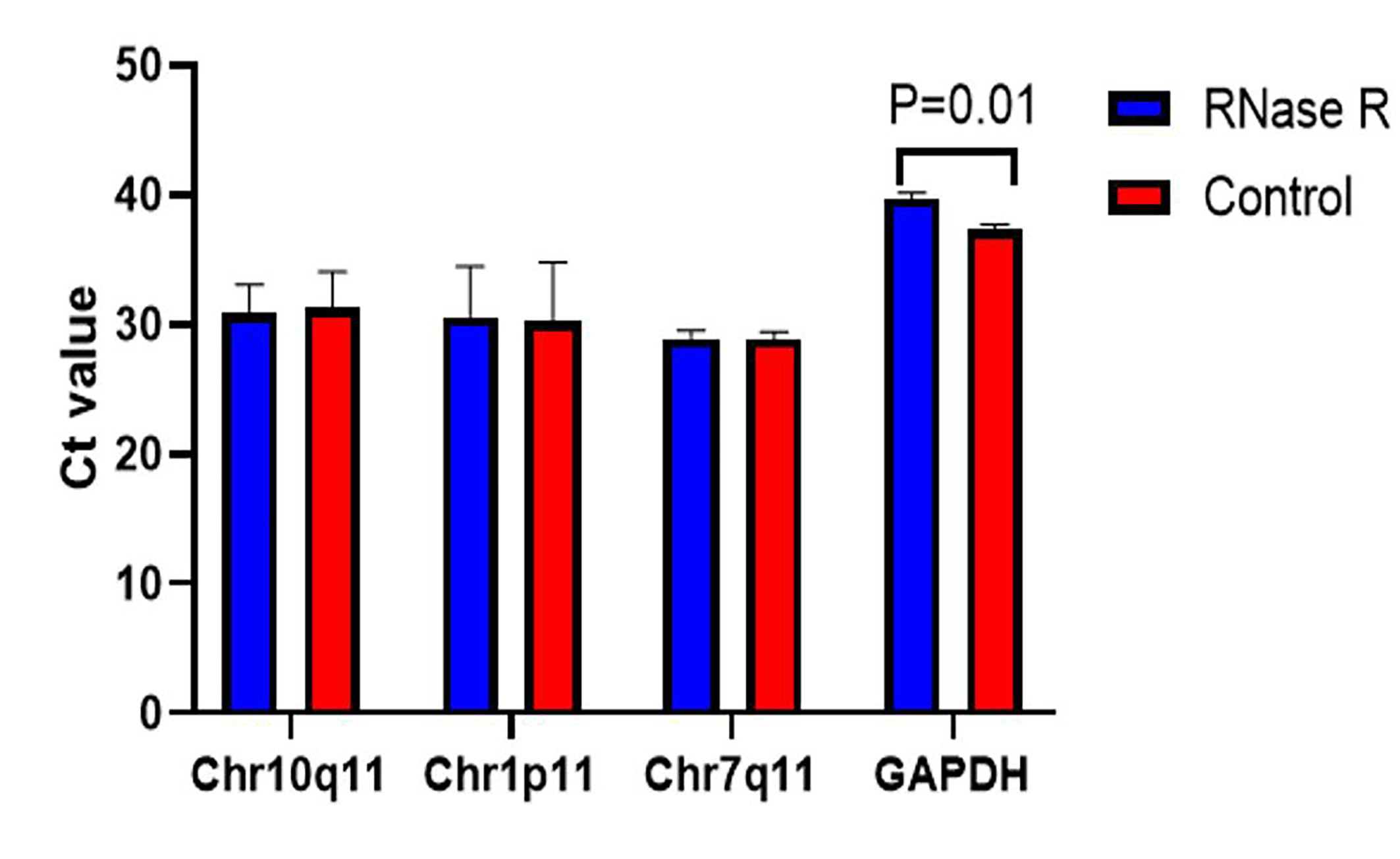

Supplement: Supplementary Figure 2 — The effect of RNase R on EV circRNAs. The expression of circRNAs Chr10q11, Chr1p11, Chr7q11 and GAPDH with or without RNase R treatment. [file Image_2.tif]

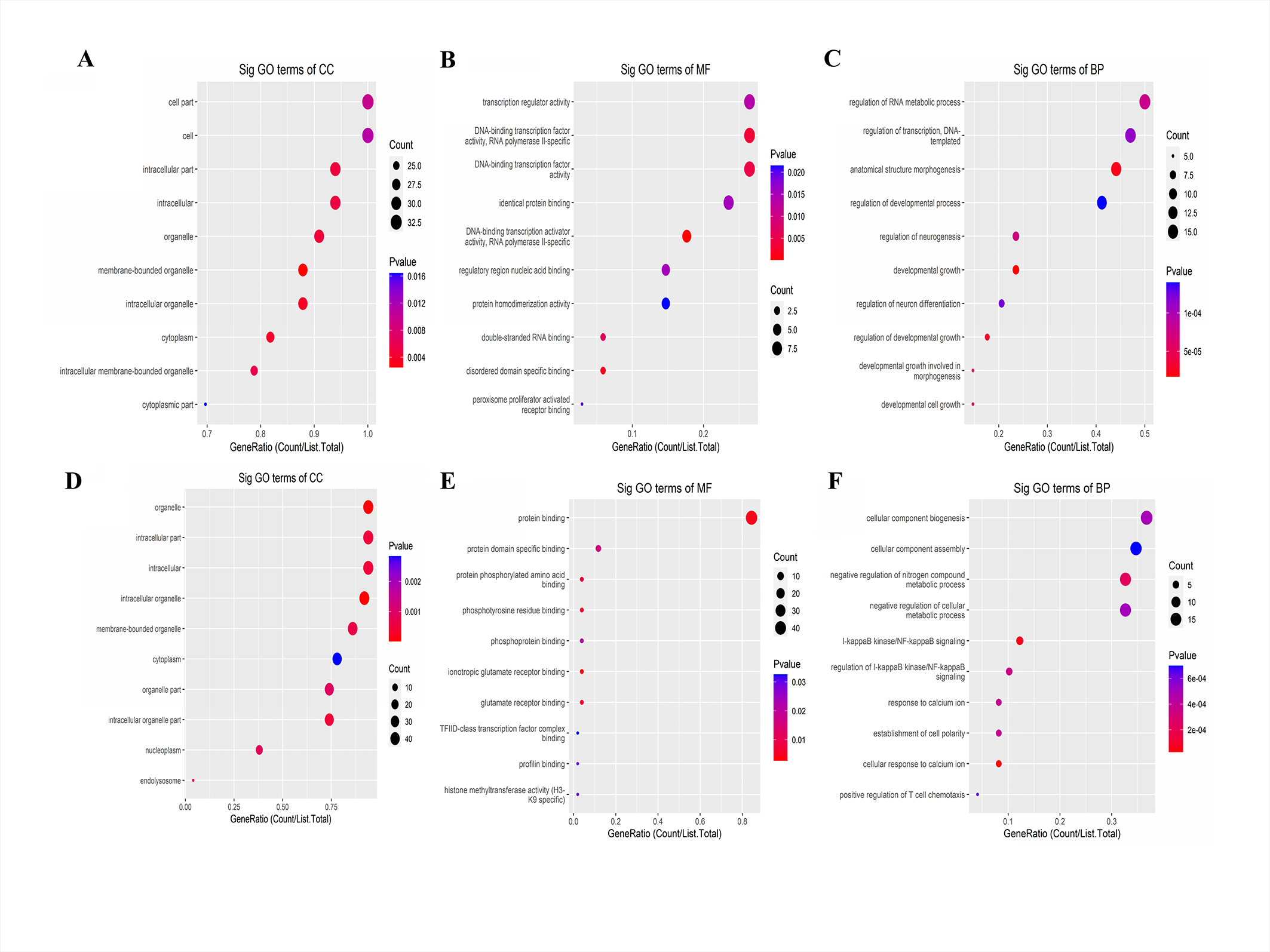

Supplement: Supplementary Figure 3 — GO analysis for mRNA from intersection. The CC, MF, and BP of the upregulated mRNAs (A–C) and the downregulated mRNAs (D–F). [file Image_3.tif]

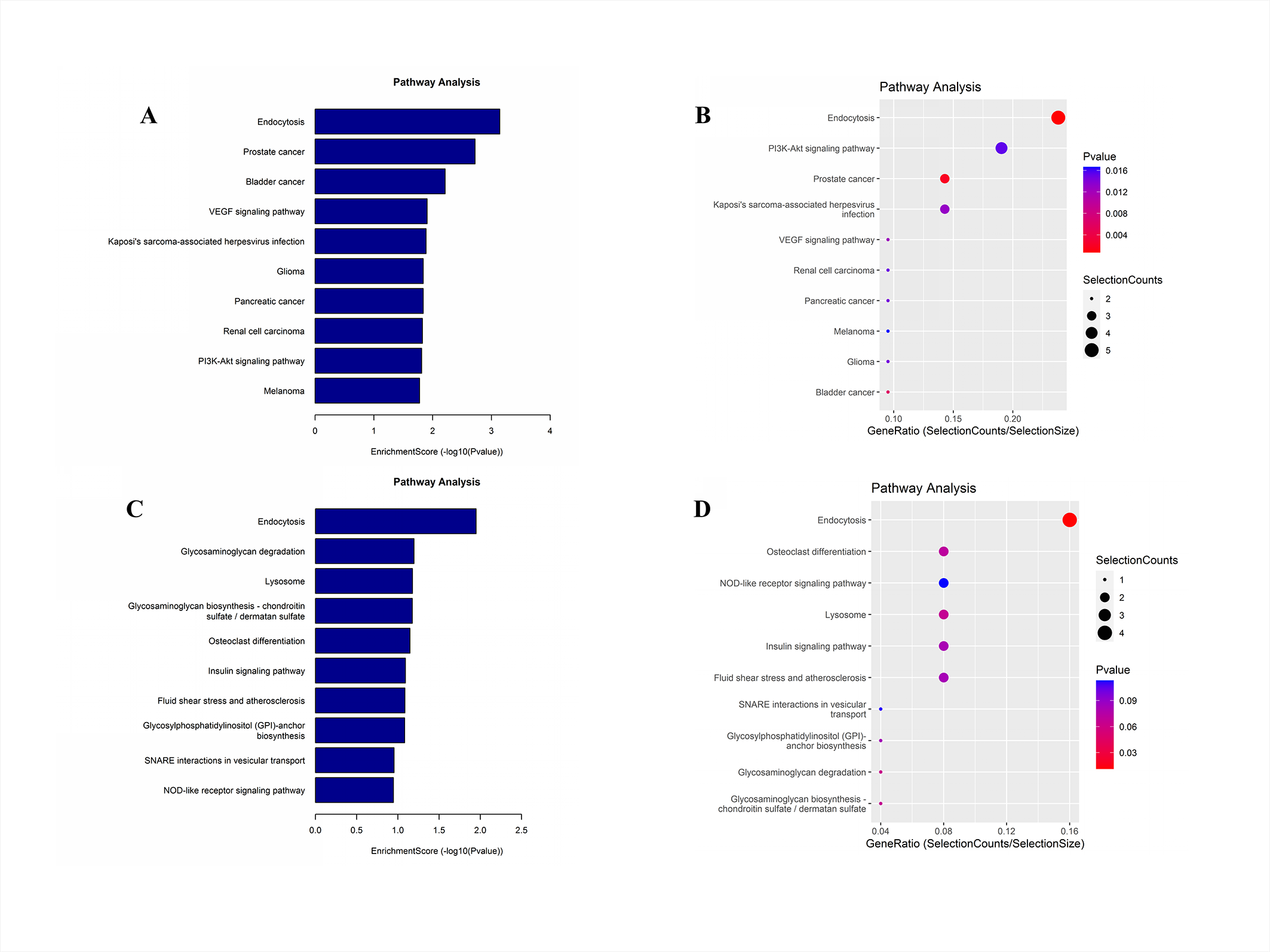

Supplement: Supplementary Figure 4 — KEGG analysis for mRNA from intersection. KEGG enrichment analysis of the upregulated mRNAs (A, B) and the downregulated mRNAs (C, D). [file Image_4.tif]
